# Three-dimensional reconstruction-guided modified arterial-based complexity scoring system for nephron-sparing surgery: comparative outcomes of on-clamp and off-clamp tumor enucleation in renal cell carcinoma

**DOI:** 10.3389/fsurg.2025.1683222

**Published:** 2025-11-06

**Authors:** Congcong Xu, Yiwei Jiang, Jiaqi Du, Kefan Yang, Qifeng Zhong, Dekai Liu, Cheng Zhang, Yichun Zheng

**Affiliations:** 1 Department of Urology, The Second Affiliated Hospital, Zhejiang University School of Medicine, Hangzhou, China; 2 Department of Urology, The Fourth Affiliated Hospital, Zhejiang University School of Medicine, Yiwu, China; 3 Department of Urology, Sir Run Run Shaw Hospital, Zhejiang University School of Medicine, Hangzhou, China

**Keywords:** nephron-sparing surgery, tumor enucleation, zero ischemia, modified arterial-based complexity, renal cell carcinoma

## Abstract

**Background:**

Various modalities of nephron-sparing surgery (NSS) exist; however, a comprehensive standard for determining the most suitable approach for specific kidney cancer patients remains elusive. This study aims to establish a novel scoring system that will assist urologists in formulating tailored surgical plans.

**Methods:**

We conducted a comparative assessment of perioperative and prognostic data for these surgical types, alongside tumor contact surface area and arterial-based complexity of 205 patients for classification and regression analysis. Finally, a modified arterial-based complexity (mABC) scoring system was developed to enhance this assessment methodology.

**Results:**

Despite no statistical differences in demographic data, we found that the off-clamp tumor enucleation (TE) group experienced greater estimated blood loss, drainage, catheterization, and longer hospital stays compared to the other two groups. However, this group also had shorter surgical times and less kidney function impairment, particularly in patients with renal dysfunction. Subgroup analysis indicated that when the mABC score was ≥4, patients in the off-clamp TE group showed significant increases in the rate of reduction in eGFR, blood loss, postoperative complications, postoperative drainage volume, and postoperative hospital days compared to patients in the other two groups.

**Conclusions:**

The findings indicate that patients with fewer challenges in renal surgery may benefit from off-clamp TE, while those facing greater difficulty may find on-clamp TE more appropriate. This distinction, based on mABC scoring criteria, emphasizes the importance of tailoring the surgical approach to individual patient needs.

**Trial Registration:**

Our study has been approved by the Ethics Committee of The Fourth Affiliated Hospital of Zhejiang University School of Medicine (K2023003) on 10/02/2023 and by clinical trials (*N*CT05790122) on 27/03/2023.

## 1 Introduction

Renal cell carcinoma (RCC) is a common tumor of the urinary system, accounting for 4.1% of all malignant tumors (1). According to the latest statistics from GLOBOCAN, there were 71,676 new cases of renal cancer and 15,259 deaths in the United States in 2021, while there were 77,410 new cases and 46,345 deaths in China (2–4). The incidence of renal cancer has increased at an average rate of 6.5% per year over the past 20 years, and the number of related deaths in the urinary system has already surpassed that of bladder cancer and is now ranked first in China (5). As renal ultrasonography is a routine component of physical examinations for individuals over 40 in China, most patients are diagnosed with localized, early-stage renal cancer (6). At present, guidelines retain nephron-sparing surgery (NSS) as the first-line treatment for early localized renal cell carcinoma (7–9). NSS includes partial nephrectomy (PN) and tumor enucleation (TE) (10). Unlike PN, TE is a surgical method of blunt separation along the natural cutting plane between the tumor capsule and healthy substance, completely stripping off the tumor tissues while preserving the normal renal tissues to the greatest (11, 12). However, partial nephrectomy remains the predominant technique, primarily due to two conceptual concerns: the risk of positive surgical margins with TE and its perceived higher technical difficulty compared to traditional PN (13, 14).

In addition, the renal artery trunk needs to be temporarily clamped to achieve a relatively bloodless operating environment to ensure the safety of tumor resection in the traditional nephrectomy process, but the warm ischemic time (WIT) is too long, inevitably affecting the function of normal renal tissue. The study showed that the shorter the WIT of the kidney, the better was the postoperative renal function recovery postoperatively (15). When the ischemia time is more than 28 min, the renal function is seriously damaged. Every minute was important when the renal artery was occluded. Therefore, minimizing the ischemia time to zero is the goal of every surgeon.

Currently, in the clinical setting, there are few people delving into the field of TE combined with zero-ischemia technology. Over the past three years, our team has performed nearly one hundred zero-ischemia renal tumor enucleations. Building on this experience, we have adopted a sutureless technique to avoid potential functional damage from parenchymal suturing and to minimize nephron loss. It is particularly important for patients with solitary kidneys or transplanted kidneys because their normal nephrons are very precious.

In this study, we reviewed patients managed for NSS to evaluate whether the surgeries would be safe, better preserve renal function, and improve rapid rehabilitation. Moreover, we established an assessment system for RCC patients to evaluate which surgical approach is suitable. Throughout this article, sutureless unclamped TE is referred to as off-clamp TE, and traditional TE is referred to as on-clamp TE.

## 2 Methods

### 2.1 Patients

**Figure 1:**
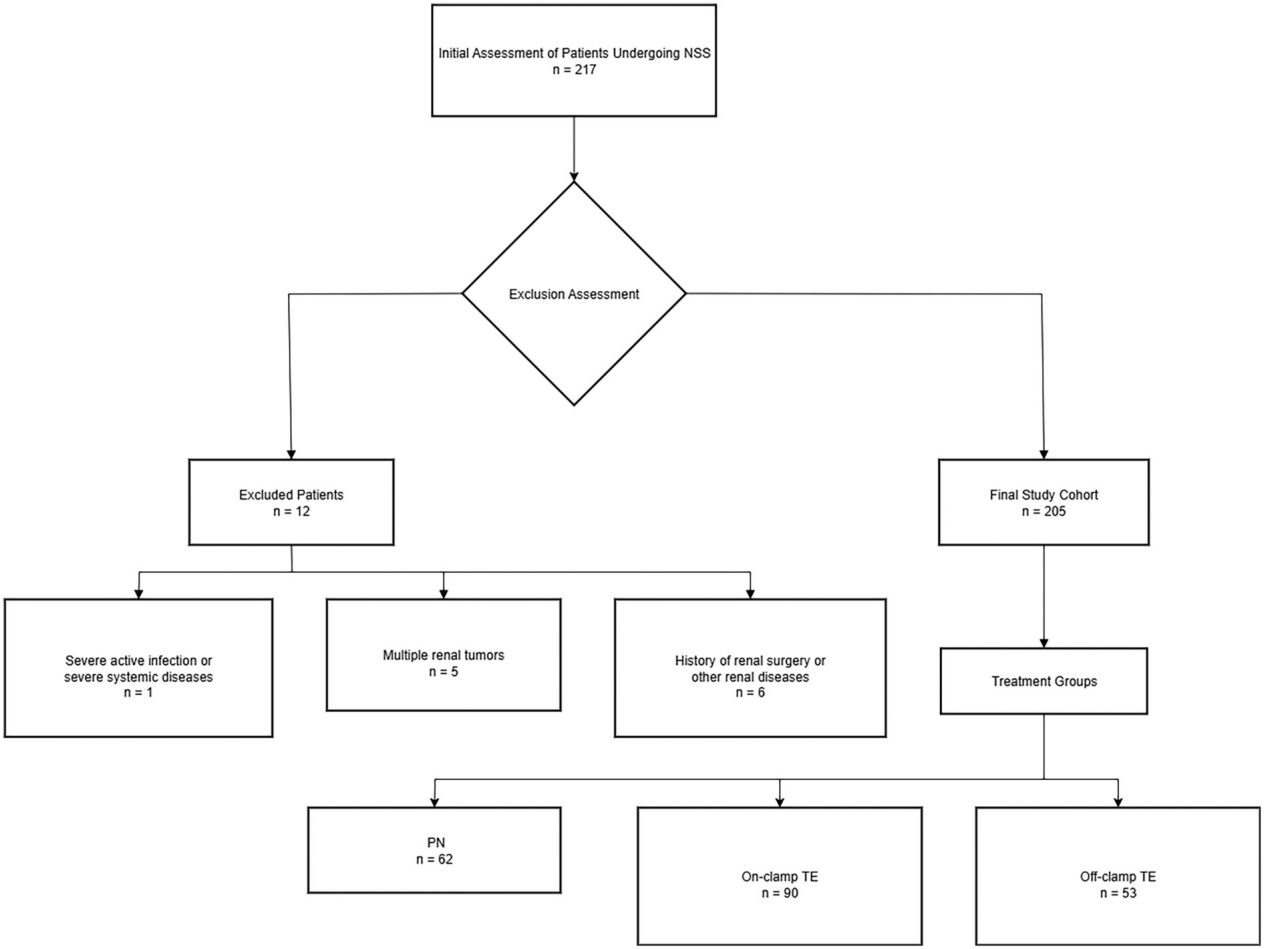
Flow diagram of patients through the study.

The study traced all patients with solid renal tumors who underwent NSS performed by one surgeon from 2017 to 2022. 217 patients were enrolled consecutively,after excluding cases that did not meet the requirements, 205 patients were selected (Figure 1). Dedicated genitourinary pathologists performed all the pathological analyses, including margin status and width. A negative surgical margin was defined as the presence of an intact tumor pseudocapsule with or without a quantifiable rim of the normal parenchyma (Supplementary Figure S1). The study followed the STROBE guidelines for reporting, ensuring the transparency and completeness of the observational research.

### 2.2 Surgical procedure

**Figure 2:**
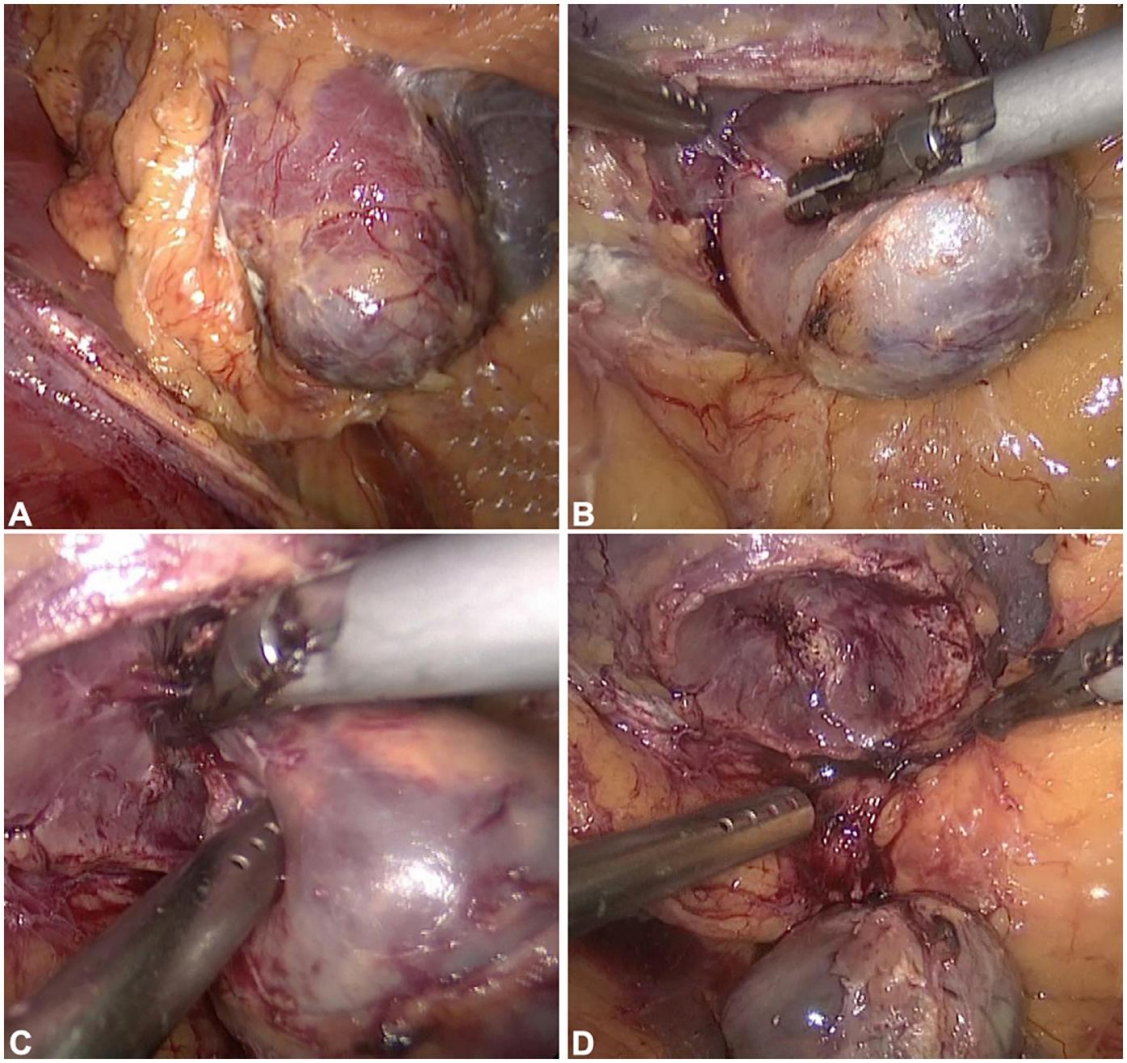
Key procedural steps in off-clamp tumor enucleation. **(A)** Tumor exposure. **(B)** Tumor resection. **(C)** Identification and coagulation of tumor-feeding vessels. **(D)** Resultant wound bed following tumor excision. As depicted, when tumors are superficially positioned and dissection is meticulously performed along the pseudocapsular plane with precise ligation of feeding vessels, intraoperative blood loss remains minimal throughout the procedure.

All surgical procedures were performed under general anesthesia by a single surgeon with 10 years of experience, and over 500 RCC surgeries were performed. PN was performed according to traditional procedures (16, 17). Off-clamp TE was performed as follows: (1) The patient was placed in a lateral position after anesthesia, and routine disinfection was performed. (2) A 1.5 cm transverse incision was made 2 cm above the iliac crest for the endoscopic port. The two access ports were located on the anterior and posterior axillary lines. The three holes form an inverted triangle. (3) Liberate the surface fat, open the fascia of G, separate the tissue around the renal pedicle, and free the renal artery. (4) The tumor was fully exposed on the renal surface, and an ultrasonic knife was used to dissect along the tumor pseudocapsule under zero-ischemia conditions. (5) Stress check, no obvious bleeding, correct counting of instruments and gauze, suturing of the incision, and completion of surgery (Figure 2; Supplementary Figure S1).

### 2.3 Contact surface area calculation

**Figure 3:**
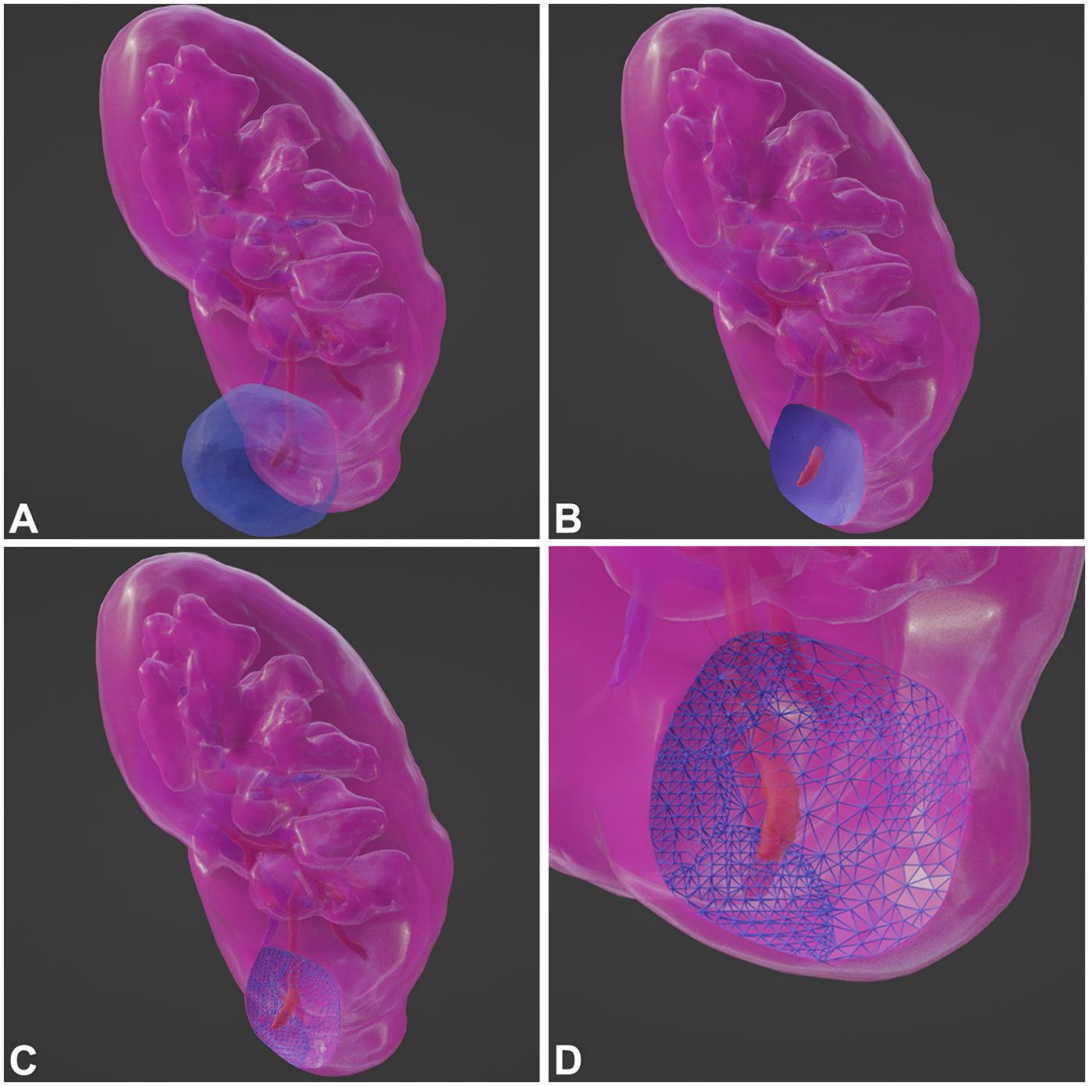
Major steps of CSA calculation. **(A)** Model. **(B)** Intersect Region. **(C)** Sub-Divide. **(D)** Zoom-in and Look.

First, it is necessary to collect the original data of renal artery-enhanced CT for each patient and then perform three-dimensional reconstruction to generate a three-dimensional representation. Similarly, three-dimensional imaging data of the tumor were obtained using medical imaging techniques. Subsequently, image segmentation algorithms were applied to extract the tumor from the image, yielding a three-dimensional tumor model. Next, the kidney organ model and tumor model were registered to ensure that they were in the same coordinate system. The contact surface between the tumor and kidney can be determined by calculating the intersection between the organ and tumor models. The contact surface area (CSA) between the tumor and kidney was quantified using surface mesh analysis of the geometric interface. This involves partitioning the contact surface into small triangular elements and summing their individual areas (Figure 3).

### 2.4 Modified arterial based complexity scoring system

Modified arterial based complexity (mABC) scoring system is a combination of the Tumor Contact Surface Area (CSA) and the arterial-based complexity (ABC) score system. The scoring method for CSA was performed as described previously. If <20 cm^2^, A score of 1 was assigned if <20 cm^2^, and a score of 2 was assigned if ≥20 cm^2^. The scoring method for ABC was the same as that described in a previous study. If the tumor was located in the renal cortex and did not invade the renal medulla, it was assigned a score of 1; if the tumor invaded the renal medulla but not the renal sinus, it was assigned a score of 2; if the tumor invaded the renal sinus or hilum, it was assigned a score of 3. Finally, these two scores were added together, with a total score ranging from 2 to 5 points (Supplementary Table S1).

### 2.5 Statistical analysis

Categorical variables are presented as frequencies and percentages. Continuous variables following a normal distribution are expressed as mean ± standard deviation (x ± s), while those not conforming to normality are described using median and interquartile range (M, IQR). The Mann–Whitney *U* test and the Kruskal–Wallis H test were used to compare two or more non-normally distributed continuous variables, respectively. One-way ANOVA was employed to compare multiple normally distributed continuous variables, with *post-hoc* pairwise comparisons conducted using the Bonferroni test. Categorical variables were compared using the Pearson chi-square test. Univariate and multivariate binary logistic regression analyses were performed to examine the associations between various clinical variables and postoperative eGFR, as well as other perioperative parameters. Depending on whether the paired sample data met assumptions of normality, homogeneity of variances, and normality of differences, either paired t-tests or Wilcoxon signed-rank tests were applied to compare perioperative estimated glomerular filtration rate (eGFR) within the study population. GraphPad Prism 10.1.2 was used to generate line graphs illustrating the relationship between mABC scores and various perioperative variables. A statistical significance level of *p* < 0.05 was adopted for all analyses. All data were analyzed using SPSS version 26.0 (SPSS Inc., Chicago, IL, USA).

## 3 Results

A total of 205 patients meeting the inclusion criteria for the study were divided into three groups based on the type of surgery they underwent. The groups were Group 1 (PN, 62 cases; 30.2%), Group 2 (on-clamp TE, 90 cases; 43.9%), and Group 3 (off-clamp TE, 53 cases; 25.9%). Baseline data of the study population are shown in Table 1. There were no statistically significant differences between the groups in terms of age, body mass index (BMI), sex, or ASA classification. There were also no statistically significant differences in the baseline renal function between the groups. The RENAL scores were 7.08 ± 1.60 (Group 1), 6.48 ± 1.60 (Group 2), and 6.77 ± 1.63 (Group 3) (*p* = 0.08). The PADUA score for Group 1 was higher than that of the other two groups (9.02 ± 1.65, 8.16 ± 1.47, and 8.57 ± 1.50, respectively; *p* = 0.004). The median tumor diameter in Group 3 was smaller than the other two groups (2.78 cm, 2.80 cm, and 2.30 cm for Groups 1, 2, and 3, respectively, *p* = 0.03). The tumor contact surface area (CSA) in Group 3 was lower than that in the other two groups (21.7 ± 17.1, 16.8 ± 10.7, and 13.2 ± 12.2, respectively; *p* = 0.003). There were no statistically significant differences between the groups in terms of ABC score (1.69 ± 0.69, 1.61 ± 0.53, 1.66 ± 0.65, respectively; *p* = 0.71) and mABC score (3.11 ± 1.13, 2.98 ± 0.98, 2.85 ± 0.91, respectively; *p* = 0.52).

**Table 1:** Patient baseline characteristics.

Parameters	Group 1	Group 2	Group 3	*p* value
Patients, *n* (%)	62 (30.2)	90 (43.9)	53 (25.9)	
Age, year, median (IQR)	54 (23–78)	53 (27–87)	56 (32–73)	0.69
BMI, median (IQR)	25.5 (18.1–35.2)	25.2 (18.7–30.8)	25.7 (21.8–31.7)	0.84
Male, *n* (%)	36 (58.1)	51 (56.7)	30 (56.6)	0.92
ASA class, *n* (%)				
≤2	54 (87)	79 (88)	48 (90)	0.83
3	8 (13)	11 (12)	5 (10)	
Renal function status				
Baseline eGFR, ml/min per 1.73 m^2^, mean ± SD	99.2 ± 15.3	99.1 ± 15.7	98.3 ± 16.9	0.83
Baseline CKD ≥3, *n* (%)	3 (4.8)	4 (4.4)	4 (7.5)	0.71
Tumor complexity				
Tumor diameter on CT, cm, mean ± SD	2.78 (1.07–5.95)	2.80 (1.03–5.26)	2.3 (1.04–6.76)	0.03
RENAL score, mean ± SD	7.08 ± 1.60	6.48 ± 1.60	6.77 ± 1.63	0.08
PADUA score, mean ± SD	9.02 ± 1.65	8.16 ± 1.47	8.57 ± 1.50	0.004
ABC score, mean ± SD	1.69 ± 0.69	1.61 ± 0.53	1.66 ± 0.65	0.71
CSA, mean ± SD	21.7 ± 17.1	16.8 ± 10.7	13.2 ± 12.2	0.003
mABC score, mean ± SD	3.00 ± 1.01	2.94 ± 0.98	2.75 ± 0.88	0.34

BMI, body mass index; ASA, American Society of Anesthesiologists; SCr, serum creatinine; eGFR, estimated glomerular filtration rate; BUN, blood urea nitrogen; CKD, chronic kidney disease; CT, computed tomography; R.E.N.A.L, radius exophyic/endophytic nearness anterior/posterior location; PADUA, preoperative aspects and dimensions used for an anatomical; ABC, arterial based complexity; eCSA, estimated contact surface area; mABC, modified arterial based complexity; SD, standard deviation; IQR, interquartile range.

**Figure 4:**
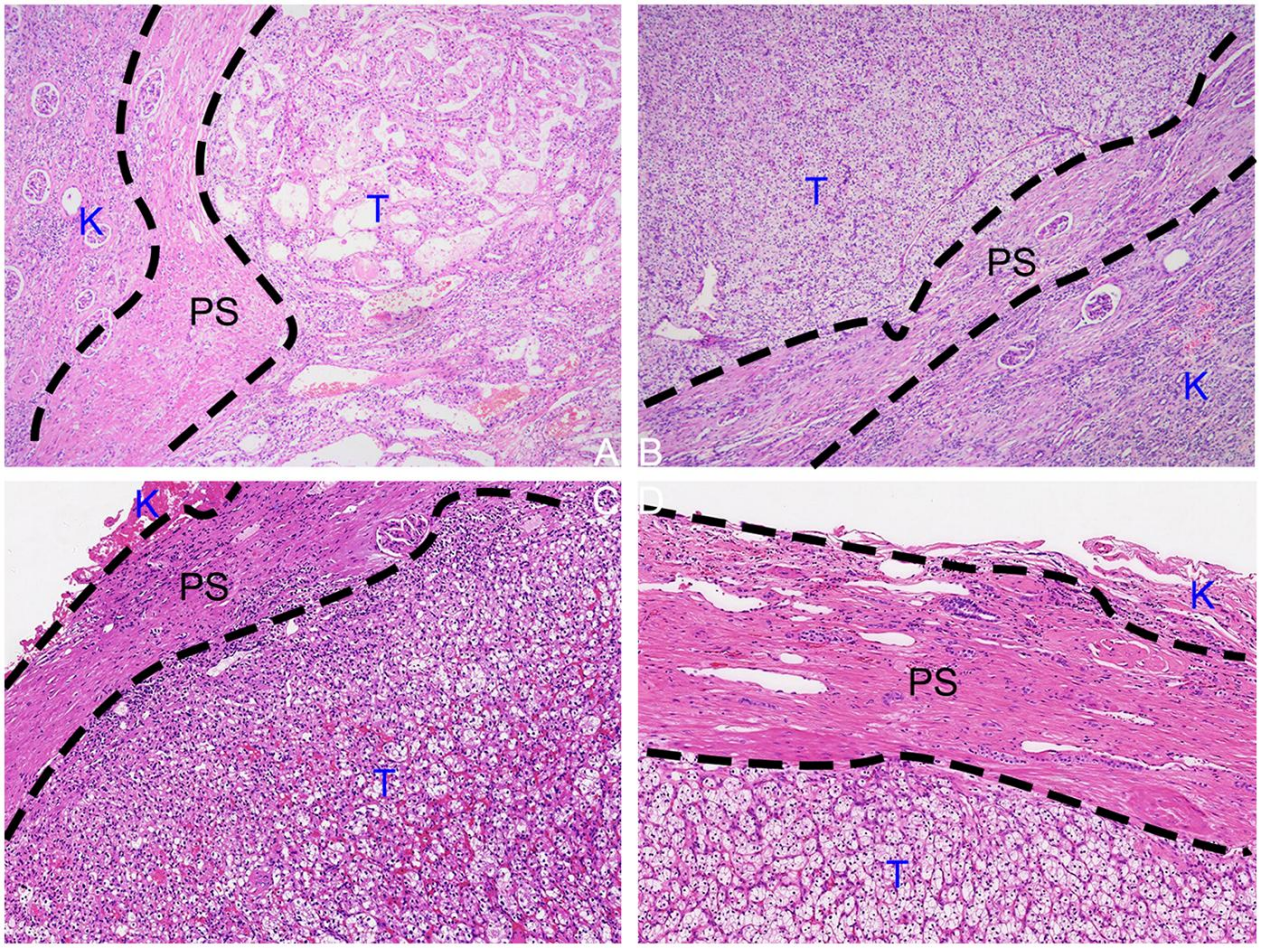
HE-stained tissue sections cut from formalin-fixed paraffin-embedded specimens from the tumor-kidney boundary. **(A,B)** PN postoperative specimen. **(C,D)** TE postoperative specimen. (K, kidney; PC, pseudocapsule; T, tumor; F, perirenal fat).

Perioperative data of the study population are shown in Table 2. There were no significant differences in the median operation time, transfusion, or positive surgical margin (PSM) rates between the groups. Group 3 had the shortest WIT, followed by Group 2, and Group 1 had the longest (37, 34, and 0 min, respectively; *p* < 0.001). Group 3 had the highest estimated blood loss (EBL), followed by Group 1, and Group 2 had the least EBL (90 ± 46 ml, 35 ± 14 ml, 253 ± 121 ml, respectively; *p* < 0.001). Group 2 had a shorter median catheterization and length of stay (LOS) (*p* < 0.001). The median drainage was lower in Groups 1 and 2 (*p* < 0.001). At 2 weeks postoperatively, Group 3 had the smallest eGFR reduction rate compared to groups 1 and 2 (4.2 ± 4.8%, 3.1 ± 3.2%, 0.9 ± 1.7%, respectively; *p* < 0.001). At 6 months postoperatively, the eGFR reduction rates for Groups 2 and 3 were smaller than that for Group 1 (6.2 ± 6.7%, 2.0 ± 3.2%, and 0.8 ± 3.0%, respectively; *p* < 0.001). Early postoperative complications were not common among the groups, with a total of 13 perioperative complications in all cases [6 (9.7%), 3 (3.3%), and 4 (7.4%), respectively; *p* = 0.264], and 3 patients experienced fever and anemia. Specifically, there were 10 cases of postoperative fever [5 (8.1%); 2 (2.2%); 3 (5.7%), respectively; *p* = 0.247], with no statistical difference; and 6 cases of postoperative anemia [2 (3.2%); 1 (1.1%); 3 (5.7%), respectively; *p* = 0.293], with no statistical difference. One patient in Group 3 required DSA intervention for embolization after surgery. Pathological findings indicated that all tumor specimens had a pseudocapsule, and none had a positive surgical margin (Figure 4).

**Table 2:** Perioperative outcomes.

Perioperative outcomes	Group 1	Group 2	Group 3	*p* value
Patients, *n* (%)	62 (30.2)	90 (43.9)	53 (25.9)	
Operative time, min, median (IQR)	104 (63–160)	100 (50–159)	90 (45–200)	0.13
WIT, min, median (IQR)	37 (24–64)	34 (17–53)	0	*p* < 0.001
EBL, ml, mean ± SD	90 ± 46	35 ± 14	253 ± 121	*p* < 0.001
Transfusion, *n* (%)	3 (4.8)	1 (1.1)	2 (3.8)	0.37
Postoperative complication, *n* (%)	6 (9.7)	3 (3.3)	4 (7.5)	0.26
Positive surgical margins, *n* (%)	0	0	0	
drainage, ml, mean ± SD	165 ± 79	132 ± 56	245 ± 115	*p* < 0.001
Catheterization, hours, median (IQR)	49 ± 16	37 ± 17	58 ± 20	*p* < 0.001
LOS, hours, mean ± SD	63 ± 19	56 ± 14	75 ± 27	*p* < 0.001
Postoperative eGFR (2 weeks), ml/min per 1.73 m^2^, mean ± SD	94.5 ± 17.0	96.4 ± 17.1	97.5 ± 17.2	0.82
Postoperative eGFR (6 months), ml/min per 1.73 m^2^, mean ± SD	93.6 ± 17.8	97.6 ± 17.3	97.5 ± 17.0	0.34
eGFR reduction rate (2 weeks), %, mean ± SD	4.2 ± 4.8	3.1 ± 3.2	0.9 ± 1.7	*p* < 0.001
eGFR reduction rate (6 months), %, mean ± SD	6.2 ± 6.7	2.0 ± 3.2	0.8 ± 3.0	*p* < 0.001
New-onset CKD ≥3, *n* (%)	0	2 (2.2)	0	0.28
Follow-up, mo, median (IQR)	32 (7–81)	24 (11–49)	23 (6–71)	*p* < 0.001
Tumor recurrence, *n* (%)	0	1	0	0.92

SD, standard deviation; IQR, interquartile range; WIT, warm ischemia time; EBL, estimated blood loss; LOS, length of stay; eGFR, estimated glomerular filtration rate; BUN, blood urea nitrogen; CKD, chronic kidney disease. eGFR reduction rate was calculated as percentage difference in actual postoperative GFR compared with predicted GFR.

Logistic regression analysis was used to explore the factors affecting renal function (Table 3). In the univariate regression analysis, patients’ age (OR 1.05, 95%CI 1.01–1.08; *p* = 0.007), preoperative eGFR (OR 0.93, 95%CI 0.91–0.96; *p* < 0.001), RENAL score (OR 1.46, 95%CI 1.14–1.89; *p* = 0.003), PADUA score (OR 1.54, 95%CI 1.18–2.01; *p* = 0.001), mABC score (OR 2.64, 95%CI 1.67–4.13; *p* < 0.001), and surgical approach (OR 5.80, 95%CI 1.59–21.20; *p* = 0.008) were significantly associated with postoperative long-term renal function decline. After removing irrelevant factors, we further conducted a multivariate regression analysis, and the results showed that: patients’ preoperative eGFR [odds ratio [OR] 0.89, 95% confidence interval [CI] 0.86–0.93; *p* < 0.001], mABC score (OR 7.48, 95%CI 2.73–20.49; *p* < 0.001), and surgical approach (OR 10.05, 95%CI 1.75–57.61; *p* < 0.001) were associated with renal function preservation in patients. In conclusion, we have three main findings: patients with abnormal preoperative eGFR, higher mABC scores, and those undergoing PN surgery experienced more severe postoperative renal function damage. Additionally, we analyzed the factors related to postoperative complications (Supplementary Table S2), and the results showed that the higher the mABC score (OR 2.93, 95%CI 1.26–6.83; *p* = 0.013) and the lower the preoperative eGFR level (OR 1.08, 95%CI 1.01–1.16; *p* = 0.024), the higher the incidence of postoperative complications. Furthermore, based on the data from Supplementary Tables S3, S4, the mABC score (OR 5.45, 95%CI 2.92–10.18; *p* = 0.001) was a risk factor for operative time, and the mABC score (OR 5.14, 95%CI 266–9.99; *p* = 0.001) and off- clamp TE were risk factors for length of hospital stay.

**Table 3:** Univariate and multivariable analyses to predict >5% decrease in eGFR.

Variables	Univariable analysis	*P* value	Multivariable analysis	*P* value
	OR (95%CI)		OR (95%CI)	
Age	1.05 (1.01–1.08)	0.007	1.01 (0.96–1.06)	0.753
Gender				
female	Referent			
male	0.98 (0.45–2.14)	0.961		
Preoperative eGFR	0.93 (0.91–0.96)	*p* < 0.001	0.89 (0.86–0.93)	*p* < 0.001
RENAL score	1.46 (1.14–1.89)	0.003	0.69 (0.28–1.67)	0.405
PADUA score	1.54 (1.18–2.01)	0.001	1.14 (0.45–2.88)	0.782
mABC score	2.64 (1.67–4.13)	*p* < 0.001	7.48 (2.73–20.49)	*p* < 0.001
Resection strategy				
PN	5.80 (1.59–21.20)	0.008	10.05 (1.75–57.61)	0.01
On-clamp TE	2.32 (0.67–8.73)	0.213	1.46 (0.25–8.67)	0.68
Off-clamp TE	Referent		Referent	

PN, partial nephrectomy; TE, tumor enucleation; R.E.N.A.L, radius exophyic/endophytic nearness anterior/posterior location; PADUA, preoperative aspects and dimensions used for an anatomical; mABC, modified arterial based complexity.

We found from the above results that the preoperative eGFR level is closely related to the postoperative outcome. Therefore, we specifically selected patients with preoperative eGFR abnormalities (≤90 ml/min/1.73 m^2^) for paired comparisons (Table 4). The results showed that postoperative eGFR levels of PN (*p* < 0.001) and on-clamp TE (*p* < 0.001) were significantly lower than preoperative levels, whereas off-clamp TE showed no significant change in postoperative long-term eGFR levels (*p* = 0.306). We further compared patients with poor preoperative renal function according to surgical method (Supplementary Table S5). At 2 weeks postoperatively, the eGFR reduction rate in off-clamp TE patients was significantly lower than that in on-clamp and PN patients (7.7%, 8.8%, and 2.7%, respectively; *p* = 0.001). At 6 months postoperatively, the eGFR reduction rate in on-clamp and off-clamp TE was significantly lower than that in PN (12.2%, 7.2%, and 2.0%, respectively; *p* < 0.001). In short, in the absence of other factors, patients with preoperative eGFR abnormalities receiving off-clamp TE surgical methods could better protect their renal function.

**Table 4:** Renal functional outcomes of patients whose eGFR lower than 90 (ml/min/1.73m2).

Parameters	Preoperative eGFR	Postoperative eGFR (6 months)	*P* value
PN, median (IQR)	70.2 ± 10.6	58.8 ± 12.8	*p* < 0.001
on-clamp TE, median (IQR)	72.0 ± 14.5	67.1 ± 15.6	*p* < 0.001
off-clamp TE, median (IQR)	67.8 ± 13.0	67.2 ± 12.9	0.306

PN, partial nephrectomy; TE, tumor enucleation; IQR, interquartile range.

**Figure 5:**
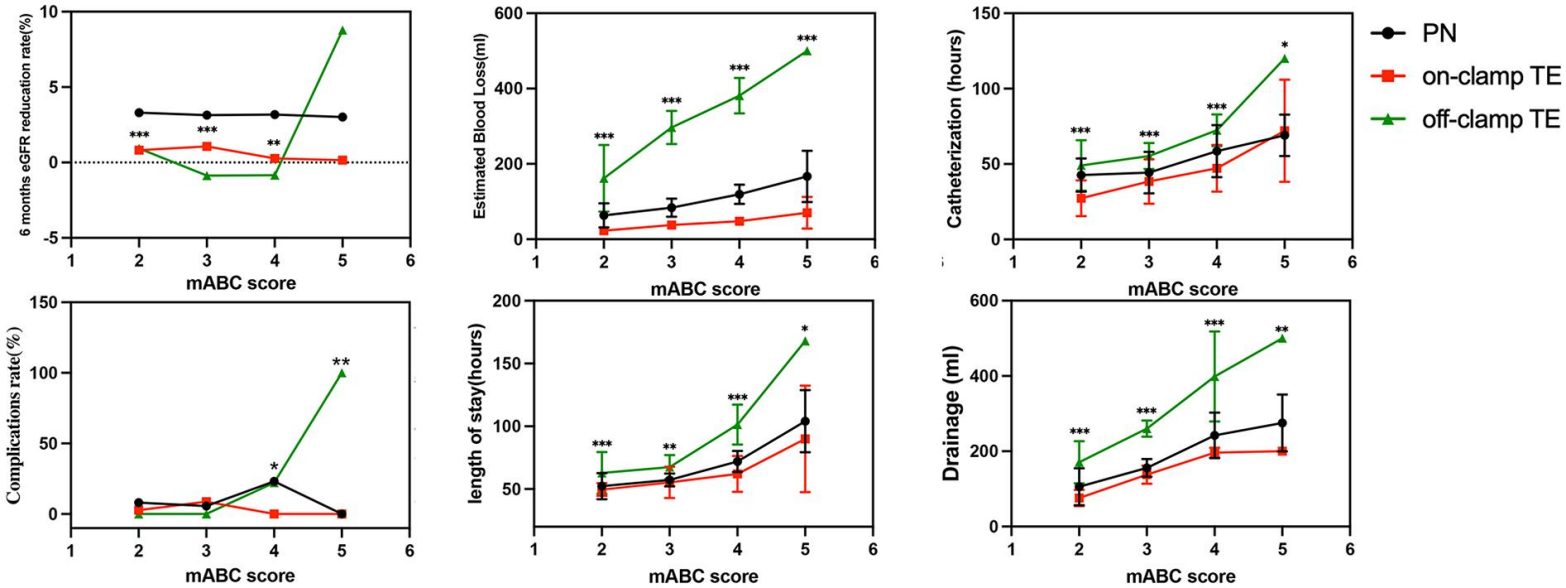
Relationship between mABC score and perioperative outcomes (*p < 0.05; **p < 0.01; ***p < 0.001).

We further studied the relationship between the mABC score and various perioperative data (Figure 5). When mABC < 4, the eGFR reduction rate in the off-clamp TE group was lower. However, when mABC ≥ 4, the eGFR reduction rate in the off-clamp TE group began to increase, and it had already exceeded that of the other two groups at five points. Similarly, when mABC ≥ 4, the off-clamp TE patients’ bleeding volume (119 ± 25 ml, 48 ± 8 ml, 381 ± 47 ml; *p* < 0.001), postoperative complications (23.1%, 0%, 22.2%; *p* = 0.029), postoperative drainage (242 ± 61 ml, 196 ± 13, 399 ± 120; *p* < 0.001), and postoperative hospital stay (72 ± 8 h, 62 ± 14 h, 101 ± 16 h; *p* < 0.001) significantly increased and were notably higher than those in the other two groups of patients. Therefore, it is preliminarily inferred that the off-clamp TE technique is more suitable for patients with an mABC score of <4. For this purpose, we conducted a chi-square test. We set a threshold mABC = 4, dividing the patients into the mABC < 4 group (*n* = 145) and mABC ≥ 4 group (*n* = 60), and found a higher probability of postoperative complications (2.1% vs. 16.7%, *p* < 0.001), surgery time ≥2 h (7.6% vs. 58.3%, *p* < 0.001), and postoperative hospital stay ≥3 days (22.8% vs. 68.3%, *p* < 0.001) in the mABC ≥ 4 group (Supplementary Table S6).

## 4 Discussion

The principle of modern minimally invasive urologic surgery is to achieve optimal therapeutic outcomes with minimal trauma (18, 19). Despite the current technological conditions allowing for the removal of only tumor tissue during nephron-sparing surgery (NSS), there is still a significant risk for certain complex renal tumors with zero ischemia partial nephrectomy (TE). Currently, no research has been conducted to guide urologists in selecting the appropriate surgical approach for NSS. With the aim of serving clinical needs, our team has summarized a large amount of data and clinical experience from previous NSS surgeries with the intention of establishing a scoring system to provide guidance for physicians when facing this choice.

As reported in the above text, off- and on-clamp TE cause less renal function damage compared to partial nephrectomy (PN), but when further comparing patients with abnormal renal function within the three groups, it was found that off-clamp TE resulted in less renal function damage than PN and on-clamp TE. Off-clamp TE, which is sutureless and has zero ischemia, results in even less renal damage, making it more suitable for patients with sensitive kidney function abnormalities. In all cases, a patient with an isolated kidney due to radical nephrectomy for contralateral renal cell carcinoma 7 years ago was considered for off-clamp TE, as he had only one functioning kidney and an eGFR of 57.1. Fortunately, postoperative kidney function did not decrease significantly (eGFR reduction rate at 6 months postoperatively was 3.9%). However, the small number of such special cases precluded meaningful intergroup comparisons.

However, concerns regarding the safety of off-clamp TE surgery exist. Therefore, based on the difficulty of surgery (mABC score), we divided the patients into groups and compared the postoperative effects of different surgical techniques. The results showed that when mABC ≥ 4, EBL with off-clamp TE began to increase dramatically. When mABC reached 5, EBL had already exceeded 400 ml, indicating a high surgical risk, which we consider unsafe. Furthermore, postoperative complications and renal function damage also began to exceed those with on-clamp TE, indicating a slower postoperative recovery. In the off-clamp cases, two patients with mABC = 5 underwent wound closure during surgery, considering the possibility of postoperative urinary fistula and bleeding. Therefore, on-clamp TE is recommended for cases with mABC ≥ 4.

In addition, two points must be considered. First, why did we choose non-assisted zero-ischemia nephron-sparing surgery (NSS) for renal cell carcinoma? Zero-ischemic NSS mainly includes assisted NSS and non-assisted NSS (20). Assisted NSS includes ablation/radiofrequency ablation-assisted zero-ischemia laparoscopic partial nephrectomy (21–24) and selective renal artery occlusion/embolization-assisted zero-ischemia laparoscopic partial nephrectomy (25–27). After reviewing the literature, we found that ablation or occlusion helps the surgeon complete the surgery under zero ischemic conditions (22–27). However, from the perspective of the medical economy, considering the poor economic conditions of many patients in China, it is necessary to reduce the cost of medical procedures in China; therefore, non-assisted NSS is more suitable for the national conditions in China. Second, most urologists do not perform ablation or occlusion operations, and if assisted NSS is performed, it often requires assistance from other departments, such as interventional radiology and vascular surgery, which makes it difficult to promote in clinical practice. Therefore, we contend that non-assisted NSS is better suited to the current clinical and socioeconomic context and is more readily adoptable in practice. Second, why do we want to introduce the mABC scoring system based on the CSA and Anatomic ABC scoring systems? CSA is a radiological parameter based on CT imaging, which numerically combines tumor size and percentage of intrarenal component (28). However, it is too simple and overlooks many parameters such as tumor location and proximity to the renal hilum (29). Although the ABC scoring system considers the complexity of the vessels involved in the operation, it cannot reflect the size of the tumor (30). The boundary between partial resection and radical resection is mostly based on a 4 cm size. We considered the advantages and disadvantages of the two scoring systems and combined them together.

Finally, it is important to acknowledge that surgical outcomes are operator-dependent. Therefore, our findings should not be construed as a rigid clinical guideline but rather as an evidence-based tool to inform surgical decision-making.

## Data Availability

The original contributions presented in the study are included in the article/Supplementary Material, further inquiries can be directed to the corresponding authors.
