# Supplementary material for: Three-dimensional reconstruction-guided modified arterial-based complexity scoring system for nephron-sparing surgery: comparative outcomes of on-clamp and off-clamp tumor enucleation in renal cell carcinoma
Source: Front Surg. 2025 Nov 6;12:1683222. doi: 10.3389/fsurg.2025.1683222 (PMC12631218; doi:10.3389/fsurg.2025.1683222)
Supplement: Supplementary file 1 [file Supplementaryfile1.docx]

Supplementary Material

# Supplementary Figures

**
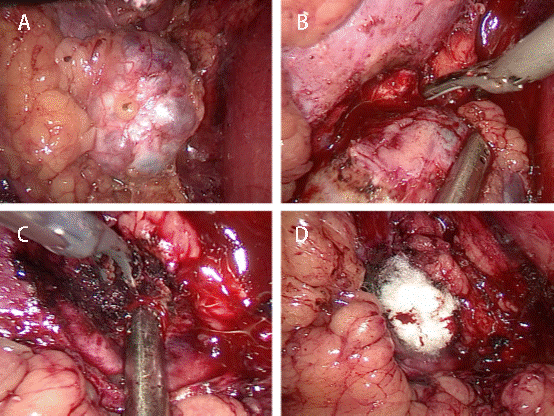
**

**Supplementary Figure 1.** Major steps of off-clamp tumor enucleation. A Tumor exposure; B Tumor resection; C Bipolar coagulation hemostasis; D Hemostatic gauze packing.

# Supplementary Tables

**Supplementary Table 1. Modified arterial based complexity scoring system**

|  | **mABC score** | | |
| --- | --- | --- | --- |
|  | 1 | 2 | 3 |
| **ABC score** | arcuate and interlobular arteries | interlobar arteries | segmental arteries or hilar vessels |
| **CSA** | <20 cm^2^ | ≥20 cm^2^ |  |
| ABC = arterial based complexity; CSA = contact surface area | | | |

**Supplementary Table 2. Univariate and multivariable analyses to predict complications**

|  | Univariable analysis |  | Multivariable analysis |  |
| --- | --- | --- | --- | --- |
| **Variables** | **OR (95%CI)** | **P Value** | **OR (95%CI)** | **P Value** |
| Age | 0.98 (0.937–1.02) | 0.369 |  |  |
| **Gender** |  |  |  |  |
| female | Referent |  |  |  |
| male | 1.75 (0.52-5.88) | 0.365 |  |  |
| Preoperative eGFR | 1.07 (1.01–1.14) | 0.018 | 1.08 (1.01–1.16) | 0.024 |
| RENAL score | 1.71 (1.17–2.50) | 0.006 | 0.67 (0.25–1.81) | 0.429 |
| PADUA score | 2.03 (1.32–3.13) | 0.001 | 2.21 (0.75-6.49) | 0.149 |
| mABC score | 4.57 (2.13–9.79) | p<0.001 | 2.93 (1.26-6.83) | 0.013 |
| **Resection strategy** |  |  |  |  |
| PN | 1.31 (0.35–4.92) | 0.687 |  |  |
| on-clamp TE | 0.42 (0.09–1.97) | 0.272 |  |  |
| off-clamp TE | Referent |  |  |  |
| PN, partial nephrectomy; TE, tumor enucleation; R.E.N.A.L, radius exophyic/endophytic nearness anterior/posterior location; PADUA, Preoperative Aspects and Dimensions Used for an Anatomical; mABC = modified arterial based complexity. | | | | |

**Supplementary Table 3. Univariate and multivariable analyses to predict OT≥2h**

|  | Univariable analysis |  | Multivariable analysis |  |
| --- | --- | --- | --- | --- |
| **Variables** | **OR (95%CI)** | **P Value** | **OR (95%CI)** | **P Value** |
| Age | 1.01 (0.98–1.03) | 0.926 |  |  |
| **Gender** |  |  |  |  |
| female | Referent |  |  |  |
| male | 1.22 (0.63-2.39) | 0.555 |  |  |
| RENAL score | 1.87 (1.46–2.40) | p<0.001 | 0.73 (0.39–1.35) | 0.312 |
| PADUA score | 2.10 (1.60–2.74) | p<0.001 | 1.80 (0.94-3.45) | 0.079 |
| mABC score | 6.61(3.76–11.61) | p<0.001 | 5.45 (2.92-10.18) | p<0.001 |
| **Resection strategy** |  |  |  |  |
| PN | 0.88 (0.39–2.01) | 0.76 |  |  |
| on-clamp TE | 0.51 (0.22–1.14) | 0.10 |  |  |
| off-clamp TE | Referent |  |  |  |
| OT, operative time; PN, partial nephrectomy; TE, tumor enucleation; R.E.N.A.L, radius exophyic/endophytic nearness anterior/posterior location; PADUA, Preoperative Aspects and Dimensions Used for an Anatomical; mABC = modified arterial based complexity. | | | | |

**Supplementary Table 4. Univariate and multivariable analyses to predict LOS≥3d**

|  | Univariable analysis |  | Multivariable analysis |  |
| --- | --- | --- | --- | --- |
| **Variables** | **OR (95%CI)** | **P Value** | **OR (95%CI)** | **P Value** |
| Age | 1.00 (0.98–1.02) | 0.98 |  |  |
| **Gender** |  |  |  |  |
| female | Referent |  |  |  |
| male | 0.90 (0.51-1.60) | 0.72 |  |  |
| RENAL score | 1.83 (1.48–2.29) | p<0.001 | 1.50 (0.83–2.71) | 0.181 |
| PADUA score | 1.83 (1.47–2.27) | p<0.001 | 1.03 (0.57-1.86) | 0.93 |
| mABC score | 2.98 (2.07–4.29) | p<0.001 | 5.14 (2.64-9.99) | p<0.001 |
| **Resection strategy** |  |  |  |  |
| PN | 0.16 (0.07–0.36) | p<0.001 | 0.03 (0.01-0.10) | p<0.001 |
| on-clamp TE | 0.10 (0.05–0.22) | p<0.001 | 0.02 (0.004-0.06) | p<0.001 |
| off-clamp TE | Referent |  | Referent |  |
| LOS = length of stay; PN, partial nephrectomy; TE, tumor enucleation; R.E.N.A.L, radius exophyic/endophytic nearness anterior/posterior location; PADUA, Preoperative Aspects and Dimensions Used for an Anatomical; mABC = modified arterial based complexity. | | | | |

| Perioperative outcomes | Group1 | Group2 | Group3 | p value |
| --- | --- | --- | --- | --- |
| Patients, n | 9 | 16 | 10 |  |
| Baseline eGFR, ml/min per 1.73 m2, median (IQR) | 72 (55.7–82.4) | 73.2 (42.8–89.9) | 71.2 (48.1–84.9) | 0.639 |
| Postoperative eGFR (2 weeks), ml/min per 1.73 m2, median (IQR) | 66.4 (39.1–75.4) | 66.3 (38.1–85.5) | 69.1 (49.6–83.1) | 0.682 |
| Postoperative eGFR (6 months), ml/min per 1.73 m2, median (IQR) | 63.1 (33.5–71.9) | 67.5 (38.8–86.2) | 71.1(50.0–84.3) | 0.337 |
| eGFR reduction rate (2 weeks), %, median (IQR) | 7.7 (3.2–31.2) | 8.8 (2.8–14.4) | 2.7 (-4.32–5.44) | 0.001 |
| eGFR reduction rate (6 months), %, median (IQR) | 12.2 (4.2–41.0) | 7.2 (2.0–12.9) | 2.0 (-5.2–4.72) | p<0.001 |
| eGFR = estimated glomerular filtration rate; IQR = interquartile range | | | | |

**Supplementary Table 5. The impact of surgical methods on renal function whose eGFR lower than 90 (ml/min/1.73m^2^)**

**Supplementary Table 6.** **Perioperative outcomes according to mABC**

| **Groups** | **Complications, n (%)** | **OT≥2h** | **LOS≥3d** |
| --- | --- | --- | --- |
| mABC<3 (n=88) | 1(1.1) | 2 (2.3) | 16 (18.2) |
| mABC≥3 (n=117) | 12(10.3) | 44 (37.6) | 58 (49.6) |
| p value | 0.010 | p<0.001 | p<0.001 |
| mABC<4 (n=145) | 3(2.1) | 11(7.6) | 33 (22.8) |
| mABC≥4 (n=60) | 10(16.7) | 35(58.3) | 41 (68.3) |
| p value | p<0.001 | p<0.001 | p<0.001 |
